# Sealing Ability of Nano-ionomer in Primary Teeth: An *ex vivo* Study

**DOI:** 10.5005/jp-journals-10005-1365

**Published:** 2016-09-27

**Authors:** Fawaz Siddiqui, Swati Karkare

**Affiliations:** 1 Lecturer, Department of Pedodontics and Preventive Dentistry, Dr. D. Y. Patil Dental College & Hospital, Pune, Maharashtra, India; 2 Professor, Department of Pedodontics and Preventive Dentistry, MGV’s KBH Dental College and Hospital, Nashik, Maharashtra, India

**Keywords:** Fuji II LC, Glass-ionomer cements, Ketac, Vitremer.

## Abstract

**Introduction:**

Microleakage is an important consideration in primary dentition because the floor of the cavity preparation may be close to the pulp. The added insult to the pulp caused by seepage of irritants around the restoration and through the thin dentin may produce irreversible pulp damage.

**Aim:**

The objective of this study was to evaluate and compare the sealing ability of three light cured (LC) resin-modified glass-ionomer cements (RMGICs) in primary anterior teeth.

**Materials and methods:**

Class V cavity was prepared on the labial surface of extracted primary anterior teeth which were then grouped and restored with Ketac N100, Fuji II LC, or Vitremer. Dye penetration test with methylene blue stain was used to record the microleakage. Depth of dye penetration was recorded in millimeters at the incisal and gingival margin using computer software.

**Results:**

The depth of dye penetration at the incisal margin in the three groups was comparable, but at the gingival margin, Vitremer showed the least dye penetration, followed by Fuji II LC, and Ketac N100. The depth of dye penetration at the gingival margin was higher than the incisal margins in all the three groups.

**Conclusion:**

Among the three RMGICs, Vitremer can be considered as the material of choice for restoring class V cavities in primary anterior teeth. Periodic recall and recare is necessary when any of the three materials are used in clinical practice.

**How to cite this article:**

Siddiqui F, Karkare S. Sealing Ability of Nano-ionomer in Primary Teeth: An *ex vivo* Study. Int J Clin Pediatr Dent 2016;9(3):209-213.

## INTRODUCTION

Microleakage is defined as the passage of bacteria, fluids, molecules, or ions along the tooth-restoration interface.^1^ This leakage may be clinically undetectable, but is a major factor influencing the longevity of dental restorations as it causes many severe biological effects on the restored tooth, including the secondary caries, pulp pathology, post-restoration hypersensitivity, and marginal breakdown.^2^ Microleakage in primary dentition is an important consideration because the seepage of irritants around the restoration and through the thin dentin may produce irreversible pulp damage.^3^

Ketac N100 restorative GIC is a nanotechnology-based paste/paste product, which compared to other resin-modified glass-ionomer cements (RMGICs) is cited by the manufacturer to be highly publishable, and easy to handle. Such a material would offer great advantages in primary anterior teeth possibly replacing the composite resin for anterior restorations with added advantage of fluoride release.

Previous studies have compared Ketac N100 with different GICs, polyacid-modified GICs, and composite resin restorations in permanent premolars, molars, and in primary molars. However, there is paucity of such studies in primary anterior teeth, which are most likely to benefit from the advantages of Ketac N100. Therefore, the aim of this study was to evaluate and compare the sealing ability by measuring the marginal microleakage in three visible light-cured (LC) resin-modified glass ionomer restorative materials in primary anterior teeth.

## MATERIALS AND METHODS

One hundred and twenty extracted primary maxillary anterior with following inclusion criteria were selected for the study: (1) Teeth with non-carious crown; (2) extracted over-retained teeth; (3) teeth extracted because of orthodontic necessity; (4) extracted teeth free from any visible developmental defects or fracture.

A box cavity of size 3 × 2 × 1.5 mm was prepared on the facial surface of each tooth in the cervical one-third, using diamond point burs in a high-speed air rotor handpiece with water coolant. The dimensions of the cavity were measured with a periodontal probe. The teeth were then randomly divided equally into three groups, namely group 1: Fuji II LC (GC, Japan), group 2: Vitremer (3M, ESPE), and group 3: Ketac N100 (3M, ESPE). The cavity was restored with the respective restorative material, which was manipulated according to the manufacturer’s instruction, particularly, where the use of primer or conditioner was indicated. All the teeth were subjected to thermo cycling of 200 cycles at temperatures of 15-35°C to 45-35°C with a dwelling time of 28 s - 2 s - 28 s - 2 s respectively. The apices of all the teeth were then sealed with self-cure acrylic resin and two coats of nail varnish were applied except for an area approximately 2 mm from the periphery of the restoration. The teeth were then immersed in aqueous methylene blue stain solution (Merck, India) for 24 hours. Following drying, the nail varnish was scraped off and the teeth were sectioned labiolingually through the center of restoration using a thin carborundum disk. The incisal and gingival restoration-tooth interface of the specimens were examined under a stereomicroscope with a magnification of 20x to measure the depth of the dye penetration in millimeters.

Statistical analysis was done using Statistical Package for the Social Sciences (SPSS) version 9 software. Analysis of variance (ANOVA) and t-test was used to compare the means of the three groups at incisal and gingival tooth-restoration interface. The confidence level was set at 95% and the level of significance (p value) equal to and/or less than 0.05 was considered statistically significant.

## RESULTS

**Figs 1A to C:**
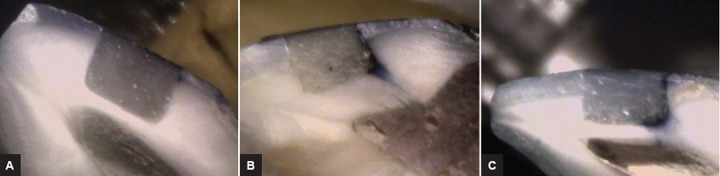
(A) Image of sample from group A; (B) image of sample from group B; and (C) image of sample from group C

Table 1 shows that at the incisal margin, the mean value of dye penetration in group 3 < group 2 < group 1, but this difference was statistically not significant (Figs 1A to C). At the gingival margin it was observed that the mean value of dye penetration in group 3 > group 1 > group 2 and this difference was found to be statistically significant. Also when the comparison of mean values of incisal and gingival surface dye penetration of each material was done, it was found that each group showed significantly higher dye penetration at the gingival margin as compared to the incisal margin (p < 0.05).

## DISCUSSION

Resin-modified GlCs are known to adhere successfully to enamel. Because chemical bonding takes place through chelating reaction with calcium on the surface of the tooth, its effect is more significant on the incisal margin.^4^ In a review of mechanism of bonding of restorative material to enamel and dentin, it was concluded that while cavity preparation in enamel margins resulted in consistently stronger bonds, unique challenges are encountered with dentin surface bonding, because enamel is 92% inorganic hydroxyapatite and dentin, that is, 45% inorganic by volume.^5^ In the present study all the groups did show microleakage at the incisal margin. This can be attributed to polymerization shrinkage that takes place in light cured resin modified glass ionomer cement (LC RMGICs).^6^ The higher filler content in the Ketac N100 (69% by weight) may have resulted not only in lower polymerization shrinkage but also in lower coefficient of thermal expansion in this material and therefore explains the least microleakage seen in this group at the incisal margin.^4^

The primer used in RMGICs plays a greater role in achieving effective bonding of teeth with RMGICs. The primer which is acidic in nature modifies the smear layer and adequately wets the tooth surface to facilitate adhesion of the material to the hard tissue.^7^ The higher microleakage at both incisal and gingival margins in teeth restored with Fuji II LC can be due to the use of dentin conditioner alone and no primer.

Microleakage at the dentin aspects of restorations remains a problem of clinical significance.^8^ The exact mechanism by which RMGICs bond to dentin is not well known.^9^ Though improved adhesion to dentin is expected because of both chemical bond from the polyacrylic acid component and formation of a hybrid layer from the hydrophilic hydroxyethyl methacrylate (HEMA), there are concerns about effect of resin component on development of ionic crosslink and subsequent marginal seal against tooth structure.^10^

**Table 1:** **Table 1:** Mean dye penetration (mm) at the tooth-restoration interface

*Margins*		*Groups*		*Mean± SD*	
Incisal margins*		Fuji II LC		0.17 ± 0.09	
		Vitremer		0.14 ± 0.07	
		Ketac N100		0.09 ± 0.06	
Gingival margins*		Fuji II LC		0.60 ± 0.41	
		Vitremer		0.25 ± 0.21	
		Ketac N100		0.95 ± 0.81	

*p-value > 0.05; ^#^p-value < 0.05

Deciduous and permanent teeth show considerable differences in the amount and distribution of mineral phase and there are also substantial differences in microstructure between them. The primary dentin is thinner than that of permanent dentin, and this has been attributed to lower bond strengths of restorative materials for primary dentin.^11^ Tubule density and diameter are also greater for primary teeth, and together, this results in a reduced area of intertubular dentin being available for bonding. Chemically, the dentin of primary teeth is more reactive to acidic conditioners, which could be explained by the reduced degree of mineralization observed for primary hard dental tissues after conditioning/primer use.^12^ The primer/conditioner in RMGICs is very acidic and this may lead to higher dentin demineralization, which can enhance dye penetration at the bonded interface. The primer has hydrophilic monomers, which also enhances water sorption and desiccation, which might explain the high microleakage at the gingival margin.

The high microleakage at the gingival margin in the present study can also be due to polymerization shrinkage which produces material shrinkage in all directions and most often at the dentin margins which are unprotected, to resist microleakage.^13^ Light cure RMGICs show polymerization shrinkage, and therefore, incremental technique has been recommended to ensure complete curing at depth and to minimize polymerization shrinkage in primary dentition.^14^

The result of the present study showed highest microleakage in Ketac N100 at the gingival margin as compared to Fuji II LC and Vitremer. This can be attributed to very superficial interaction of Ketac N100 with dentin and enamel.^15^ The bonding of Ketac N100 was found to be more of micromechanical adhesion than true chemical bonding. Therefore, micromechanical interlocking is limited to the surface roughness induced by diamond burs during cavity preparation.^16^ This explains the low microleakage in Ketac N100 at the incisal margin. The lower microleakage of Fuji II LC and Vitremer compared to Ketac N100 can be explained by the lower amount of resin in Fuji II LC in the final set restoration which is 4.5 to 6%.^17^ The final resin content in Ketac N100 has not been reported.

Ketac N100 contains monomer and a photo-initiator in its primer; it may form a resin coating on the dentin surface prior to the application of restorative material. Consequently, Ketac N100’s primary bonding mechanism is micromechanical adhesion.^18^ The very acidic Ketac N100 primer (pH = 3) may cause over demineralization of primary dentin leading to weak adhesion. Also this combination of fluoroaluminate silicate glass, polyalkenoic acid, and water in Ketac N100 restorative is responsible for the ionic glass ionomer reaction to take place very slowly over time, therefore delaying the chemical adhesiveness. The exact mechanism by which the Ketac nanoprimer treats and/or removes smear debris is still not clear.

Fuji II LC in the present study showed less leakage than Ketac N100 at the gingival margin. This is in accordance to the finding of Coutinho (2009) who reported that the Ketac N100 showed 100% adhesive failure both at enamel and dentin even when primer was used while Fuji II LC showed more (79%) cohesive failure. The same study reported that Fuji II LC showed higher bond strength to dentin as compared to Ketac N100 which was statistically significant.^15^

The role of hybrid layer or absorption layer or gel phase between cement and tooth structure is uncertain, although it has been linked to good marginal adaptation to dentin.^19^ As believed earlier that no hybrid zone/gel phase was present between Ketac N100 and apparently unaffected dentin, a very thin filler-free zone at the nano-RMGI interface was discovered which resembled the absorption layer. This filler-free zone most likely represented remnants of the primer that did not polymerize due to the presence of oxygen, and therefore only showed as a thin, sometimes non-homogeneous, layer.^15^ This layer may lead to increased microleakage due to water sorption or drying and therefore may explain the high microleakage in Ketac N100 in our study.

An independent evaluation reported that Ketac N100 contains more resin than other RMGIC materials do and that its acid-base reaction rate was lower than that of competitive products.^20^ The least microleakage in Vitremer at the gingival margin can be explained by the fact that its primer in addition to being simple mixture of HEMA with polyalkenoic acid is also modified by the attachment of polymerizable methacrylate side groups.^21^ This makes the Vitremer tri-cure RMGICs.

The present study shows that the Fuji II LC showed higher dye penetration compared to Vitremer both at incisal and gingival margin, including in its own matched teeth. This could be explained by the higher coefficient of thermal expansion of Fuji II LC (31.5 ppm/°C) as compared to Vitremer (11.5 ppm/°C).^22^ This means that Fuji II LC is more susceptible to thermal stresses as compared to Vitremer and hence in the present study showed higher leakage than Vitremer.

Curing shrinkage appears within 5 minutes after polymerization and proceeds for next 24 hours.^23^ This shrinkage gives rise to contraction stress which can damage the adhesive interface and create marginal gaps. The same study compared Vitremer and Fuji II LC and found that the curing shrinkage of Fuji II LC (-3.9 vol. %) was higher than Vitremer (-2.4 vol. %) both at 5 minutes and 24 hours. Also the authors found that volumetric change and water content of Fuji II LC (5.1 vol. %) was higher than Vitremer (2.9 vol. %) after 14 days. This would again explain the higher dye penetration in Fuji II LC as compared to Vitremer.

Gladys^24^ showed in their scanning electron microscopic (SEM) section of tooth-restoration interface that in Fuji II LC there was a thin hybrid-like structure of about 500 nm with the restorative material being only separated from the underlying dentin substrate by a thin resin-rich area. The conditioner used for Fuji II LC had caused superficial demineralization of dentin and exposure of the collagen fibril network with interfibrillar micro porosities. The hybrid layer was then formed by inter-diffusion of monomers in the interfibrillar channel. In Vitremer there was no hybrid-like zone between restorative material and tooth structure. The primer of Vitremer did not remove the smear layer nor unplug the dentinal tubules. Vitremer primer was able to modify the smear layer to permit a closer interaction of Vitremer restorative material and the dentin surface.^25^ This may explain more of chemical adhesion of Vitremer than secondary micromechanical adhesion and hence lowest microleakage observed in the gingival margin in the present study.

Since RMGICs and tooth structure bond via both mi-cromechanical interlocking and chemical polyalkenoate-hydroxyapatite bonding,^26^ it is conceivable that a greater glass ionomer character in the RMGIC may increase the chemical bonding nature, thereby increasing the bond’s durability.^27^ Though, this remains highly speculative,^6^ it explains the results of the present study. The higher resin content of Ketac N100 would limit the acid-base chelation reaction to a higher extent than Vitremer and Fuji II LC, thereby relying more on micromechanical adhesion than chemical adhesion. In the gingival margin reverse is desired. The Ketac N100, therefore, showed highest microleakage compared to Vitremer and Fuji II LC. Attin et al (1995)^23^ reported that Fuji II LC undergoes hygroscopic expansion which may reduce the leakage.^23^

Microtensile dentin bond strengths increase when bur prepared dentin is treated with the respective polyacrylic acid solution prior to insertion of Fuji II LC.^28^ Although Ketac N100 bonds to dentin *in vitro,* the bonding efficiency of Fuji II LC as measured with the microtensile bond strength test was found to be superior by 50%.^15^

A study by Gjorgievska et al^29^ showed that marginal adaptation of RMGICs in deciduous teeth was slightly inferior to that in immature permanent ones. The RMGICs tended to cause cracks adjacent to the interfacial region, but not in the material itself.^30^ Dentin consists of approximately 30% organic substance, and this contracts during drying, causing the dentin to fracture.^31^ This creates two problems in primary dentin. One, conditioning the tooth prior to placement of GIC may inhibit the development of the ion exchange layer and leaving the smear layer undisturbed complicates the development of an adhesive layer. In the same study, authors found conditioned samples were found to be better attached to the tooth surface, though this had the adverse effect of causing microcracking of the enamel.

Though this study had an experimental design and was conducted in controlled environment, it suffers the limitation of being an *ex vivo* study. Dehydrated dentinal tubules in extracted teeth may have influenced the interaction of the dentin primer and effect of dentin conditioner on the bonding of restoration to the tooth structure. The authors recommend that further study is required to determine the effect of thickness of enamel on dye penetration in cavities restored with Ketac N100.

## CONCLUSION

Vitremer is the material of choice in restoring class V cavities in primary anterior teeth. All the material tested show microleakage particularly in the gingival margin, therefore recall and recare after restorative phase of treatment planning with emphasis on prevention of caries is essential in clinical practice.
